# Locating single-point sources from arrival times containing large picking errors (LPEs): the virtual field optimization method (VFOM)

**DOI:** 10.1038/srep19205

**Published:** 2016-01-12

**Authors:** Xi-Bing Li, Ze-Wei Wang, Long-Jun Dong

**Affiliations:** 1 School of Resources and Safety Engineering, Central South University, Changsha 410083, China

## Abstract

Microseismic monitoring systems using local location techniques tend to be timely, automatic and stable. One basic requirement of these systems is the automatic picking of arrival times. However, arrival times generated by automated techniques always contain large picking errors (LPEs), which may make the location solution unreliable and cause the integrated system to be unstable. To overcome the LPE issue, we propose the virtual field optimization method (VFOM) for locating single-point sources. In contrast to existing approaches, the VFOM optimizes a continuous and virtually established objective function to search the space for the common intersection of the hyperboloids, which is determined by sensor pairs other than the least residual between the model-calculated and measured arrivals. The results of numerical examples and in-site blasts show that the VFOM can obtain more precise and stable solutions than traditional methods when the input data contain LPEs. Furthermore, we discuss the impact of LPEs on objective functions to determine the LPE-tolerant mechanism, velocity sensitivity and stopping criteria of the VFOM. The proposed method is also capable of locating acoustic sources using passive techniques such as passive sonar detection and acoustic emission.

Passive location techniques, in which signals travel from a source whose location is estimated to sensors whose positions are known, is widely used in different areas. Researchers employ passive location techniques for whale tracking^1^,^2^, structural health monitoring^3^,^4^,^5^, seismic/microseismic source inversion^6^,^7^,^8^,^9^, and seismic tomography^10^,^11^,^12^,^13^. Microseismic source location is a typical application of the passive location technique and, in conjunction with other geophysical tools such as muography^14^, can potentially provide valuable information about the lithosphere. Because of the characteristics of the received signals, the microseismic source location techniques in local monitoring operations generally use *P*-wave or *S*-wave or both arrival times to locate sources. Although several advanced picking-free techniques have been proposed, such as the source-scanning algorithm (SSA) by Kao and Shan^6^, the envelope stacking-based method by Gharti *et al.*^15^, the waveform coherence analysis by Grigoli *et al.*^16^, and many others^17^,^18^, these methods use a whole or partial seismic waveform and are therefore time consuming. Moreover, due to the complexity of the location procedure, these non-standard picking-free techniques often use grid-searching algorithms instead of the fast local searching algorithms, which can further decrease the location efficiency and resolution.

The picking quality of arrival times directly affects the accuracy of source location. At local scale, the near-field effects that exist in the seismic wavefield cannot be ignored, and therefore, the *P*-wave and *S*-wave are often intertwined^19^. This issue complicates picking and identifying seismic phases. Using current techniques, *P*-wave arrival times can be accurately picked for relatively high signal-to-noise ratio signals, such as the STA/LTA method^20^ and the higher order statistics method^21^; however, reliable picking of the *S*-wave is still problematic for local events in which the *P* coda overlaps with the *S-*wave^16^. Considering the low signal-to-noise ratio, even the picking of the *P*-wave is not satisfactory because the beginning of the *P*-wave may be concealed by the noise. Practical applications have also reported that sensors are likely to be triggered by *S*-waves but are wrongly assigned *P*-wave velocities^22^. These cases illustrate that the arrival times of both *P-* and *S*-waves may contain large picking errors (LPEs), especially when using automated picking programs.

LPEs can lead to dramatically inaccurate locations using existing location methods^23^ because the LPEs contribute to the final location result^24^. To be of practical value in an industrial environment, the microseismic monitoring system should produce information that is both reliable and timely^25^,^26^. However, it is impossible to improve the picking quality using manual methods, especially when a large number of events are received every day. The difficulty in arrival time picking and the harm of accumulated LPEs has motivated efforts to develop picking-free techniques. In addition, developing an LPE-tolerant picking-based location method is another strategy to address this issue and is crucial for automated local source locations.

The existing picking-based source location algorithms typically utilize forward modelling and iterative estimation techniques to determine the optimal location by globally minimizing a predefined objective function in the three-dimensional solution space^26^. The objective function is usually defined as the residuals between the theoretical and observed arrival times of the main seismic phases^16^. Many commonly used methods follow this procedure, such as Geiger’s method^27^ and the double-difference algorithm^28^. Various iterative techniques have been introduced to search for the optimum solution, including the simplex algorithm^29^, differential evolution algorithm^30^, genetic algorithm^31^, and LSQR algorithm^32^,^33^,^34^. In most cases, L1 and L2 norms are adopted to define the residuals. Although the L1 norm is strongly recommended by many researchers for its relative insensitivity to LPEs^29^,^35^,^36^, both L1 and L2 methods may lead to possible mislocations when the input data contain LPEs^23^,^24^.

An LPE-tolerant location method should not only be able to eliminate the solution distortion caused by LPEs but should also have the ability to send a warning when the arrival times contain too many LPEs rather than producing an unreliable solution. In this paper, we design a new method called the virtual field optimization method (VFOM) to meet these requirements. Compared with traditional methods, the VFOM searches the 3D-space for the common intersection of the hyperboloids determined by station pairs. As only the hyperboloids related to LPEs deviate from the source and the remaining hyperboloids still intersect at the source, the VFOM can eliminate the location error caused by LPEs. More importantly, by maximizing a continuously differentiable objective function (the so-called virtual field), the VFOM can be easily introduced into standard monitoring systems.

## Results

### Synthetic tests

We use synthetic tests to verify the performance of the VFOM. Because of the non-repeatability and non-verifiability of real events in an opaque medium, it is difficult to measure the error between calculated and real sources. Therefore, synthetic tests, with controllable errors in their input data, such as arrival times and velocity structures, are flexible when comparing the performance of location methods under different conditions. Specifically, we arrange a 400 m × 400 m × 400 m cubic array with 8 sensors (or stations) at its corners to receive signals. Two “real sources”, one inside the array and the other outside the array (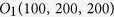 and 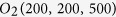, respectively), generate the microseismic signals. To simulate the uncertainty of the velocity along different paths, the velocity of each path is generated randomly in the range from 4875 to 5125 m·s^−1^. In addition, to simulate the small systemic picking errors, an extra random error term from −2 ms to 2 ms is added to each arrival time. In the case of measured arrival times containing LPEs, a dramatic error (±100 ms), which is far larger than the systemic picking error, is added to the arrival times with different probabilities. Thus, the simulated arrival times consist of velocity uncertainty, systemic picking errors and different LPE probabilities.

Four traditional location methods are applied for comparison with the VFOM. The objective functions of these four methods are given by equations (1, 2, 3, 4). For the sake of brevity, we define the four methods as TL2, TL1, DL2 and DL1, respectively. The TL2 and TL1 methods both use the residuals between the observed and theoretical arrival times to define their objective functions, and the only difference between them is that TL2 uses L2 norm whereas TL1 uses L1 norm. Differing from TL2 and TL1, DL2 and DL1 minimize the residuals between the observed and theoretical travel-time differences of station pairs. DL2 and DL1 are similar to the double-difference method in the form, but in fact are very different as DL2 and DL1 use the station pairs while the double-difference method uses event pairs^28^.


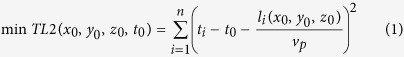



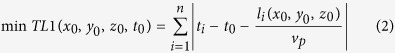



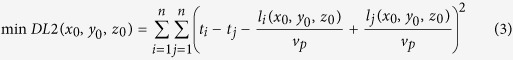



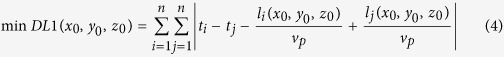


where 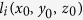 is the distance between the source and the *i*^th^ triggered sensor, 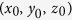 are the coordinates of the potential source, *v*_*p*_ is the constant propagation velocity, and *n* is the number of triggered sensors.

The quasi-Newton algorithm, one of the most efficient and effective algorithms for solving unconstrained optimization problems, is employed to search for the optimum solutions. As is well known, when the objective function has more than one peak, local searching algorithms may converge to local solutions if improper initial values are settled. Thus, the result depends on the initial value. To eliminate this dependence, 50 initialize-search tries are made in each location process, and the solution with the least objective value is selected as the final solution. Like many optimization algorithms requiring derivatives, the searching algorithm used in this paper is time saving. For instance, one may require several hours for a grid searching process^16^ but less than 1 second for an entire VFOM process using a popular PC machine.

**Figure 1:**
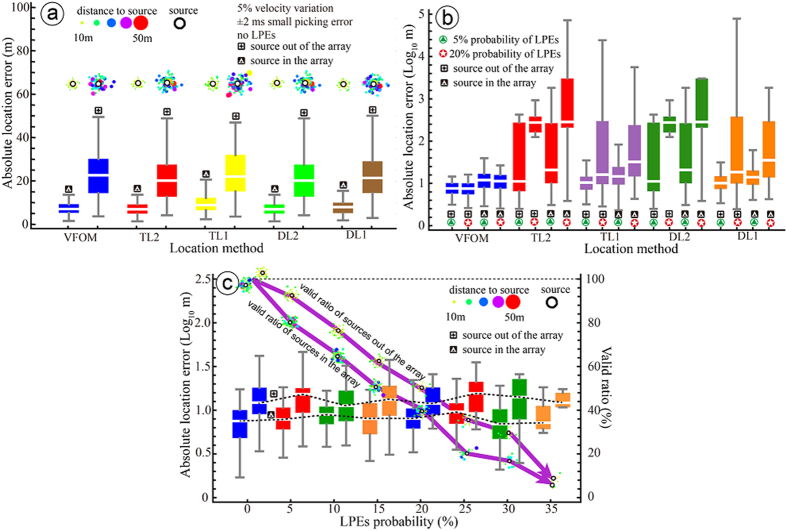
Results of synthetic tests. (**a**) Is the comparison of location errors of the VFOM and the four traditional locators using arrival times with only a 5% velocity variation and a ±2 ms small picking error. (**b**) Is similar to (**a**) except an additional 5% and 20% LPEs are added to the arrival times. (**c**) Is the location errors and location valid ratios of the VFOM using arrival times that are contaminated by different probabilities of LPEs. Stopping criterion A (*SC-A*) is used for the location process of the VFOM. The colour version of this figure is available only in the electronic edition.

To obtain reliable statistical conclusions, we repeat each event location process by changing the picking errors and the propagation velocity along each path randomly 100 times and then collect the absolute location errors. The results of the 100 simulations without LPEs are shown in the boxplots in Fig. 1a. The location errors for both the VFOM and traditional methods are very similar for both the in-array and out-array sources. In other words, the VFOM performs as well as traditional location methods when no LPEs are present in the arrival times. However, the performance of the VFOM differs from that of traditional methods in the presence of LPEs, as shown in Fig. 1b. The VFOM can still locate accurately and stably, whereas the results from the traditional methods become unreliable. For example, the average location error obtained by the VFOM stays 10~20 m while that of traditional methods reaches to hundreds even thousands of meters. These synthetic tests demonstrate that for a single-source location using arrival times with LPEs, the VFOM, due its stability and accuracy, performs better under these circumstances.

Moreover, the VFOM offers a self-evaluation strategy for picking quality by calculating the valid ratio, which is defined as the percentage of events successfully located using stopping criteria A (*SC-A*, as described in “Methods”). The valid ratio is the proportion of the optimized objective values greater than the pre-determined threshold. The relationship between the valid ratio and the probability of LPEs is displayed in Fig. 1c. Clearly, the valid ratio declines rapidly as the probability of LPEs increases. This condition offers an easy way to estimate the picking program’s quality, i.e., a higher valid ratio indicates better pickings.

### In-site explosion events

**Figure 2:**
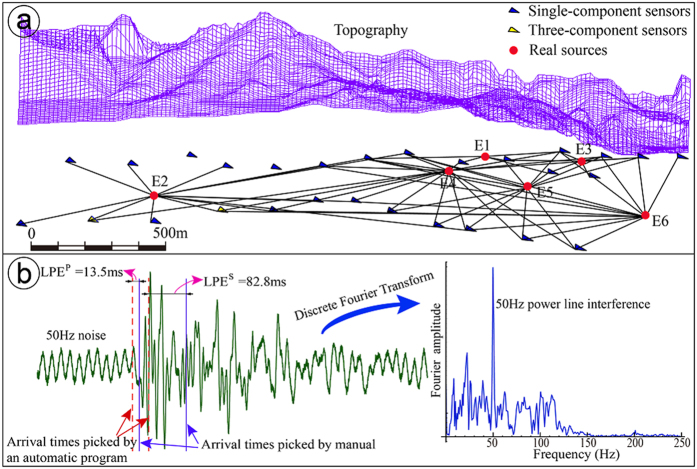
Geometry of the sensor array of the local microseismic monitoring system and a typical signal obtained by the system. (**a**) The array is set under the topography with a spatial span of 2370 m × 760 m × 200 m. The array consists of 26 single-component sensors and 2 three-component sensors. Six explosion events are shown as red points. The line between a real source and a sensor indicate that the event on one side has triggered the sensor on the other side. (**b**) A typical signal with low energy and signal-to-noise ratio. The arrival times picked by automatic programs contain LPEs due to noise, such as that from power line interference.

The data are obtained from a rock phosphorous ore mine located in Guizhou Province, China. After approximately 50 years of excavation, the mining depth has reached approximately 500–800 m below the ground surface. The high *in situ* stresses lead to a series of engineering problems, such as difficulties in the rock support of the main laneway and rock fall, spalling, slabbing and floor heaving in the permanent laneways after the installation of support^37^. A microseismic monitoring system including 26 single-component sensors and 2 three-component sensors are built to monitor microseismic events (Fig. 2a). We performed explosions of the emulsion explosive 6 times to test the proposed method. These explosion positions were measured as real sources.

The frequencies of the received signals ranged from 0 to 200 Hz. The sampling frequency was set to 6000 Hz to cover the signals’ frequency domain without distortion. In fact, many factors can affect the picking quality of arrival times in practical applications. For example, a 50 Hz power line interference can lead to LPEs both in *P*-wave picking and *S*-wave picking, especially for arrivals with low signal-to-noise ratios, as shown in Fig. 2b. To solve this problem, an LPE-tolerant location method was investigated.

Using the method proposed in this paper, we located the 6 explosion events conducted during August 20–22, 2014. For each event, we used both VFOM and traditional methods to obtain comparable results. The Quasi-Newton algorithm was employed as the searching process for all the location methods. The arrival times of the *P*-wave and the *S*-wave were picked manually to ensure a high picking quality. Furthermore, additional LPEs were added to a small part of the manually picked arrival times to simulate the picking deviation caused by automated programs. We set the *P*-wave and *S*-wave velocities to 5200 m·s^−1^ and 3300 m·s^−1^, respectively, after attempting several times to obtain the best velocity structure for all of the location methods. The stopping criterion B (*SC-B*) was used to ensure that the VFOM could always locate the source.

Table 1 shows the location errors for both the VFOM and traditional methods for the 6 explosion sources. As the location results from input data with LPEs are typically much worse than those from input data without LPEs, the methods using the L2 norm, i.e., DL2 and TL2, are obviously susceptible to LPEs. Compared to DL2 and TL2, DL1 and TL1 perform much better in terms of LPE tolerance, as the results obtained with LPEs were as accurate as those without LPEs using both *P-* and *S*-waves. However, these two methods are still too sensitive to LPEs in scenarios in which the picking of the *S*-wave is problematic and only *P*-wave arrival times are available. The VFOM stands out from these location methods because of its stable performance in both the *P*-based location and the *PS*-based location, as shown in Table 1. The location errors that use input data with LPEs are quite similar to those that use input data with LPEs using *P* arrival times only, making LPEs an unlikely cause of extra location errors. Regarding *PS*-based locations, the VFOM can locate at the same position with or without LPEs because the *S*-waves increase the number of hyperboloids intersecting at the source, which can make the location more stable. Additionally, we display the location errors for the 6 explosion events using two migration-based methods in Table 1. The picking-free migration-based method utilizes the passive kurtosis derivative waveform and the migration process to search for the source^17^. Because the passive kurtosis derivative waveform fails to clearly characterize the arrival time of the waveform for the signals, the result of the picking-free migration-based method was unsatisfactory in our case. We then manually generated a pulse at each arrival time to replace the passive kurtosis derivative waveform and execute the migration process. No significant improvement was achieved in the location result compared to the VFOM (shown in the last row of Table 1).

The stability of the VFOM under the wrong velocity model, e.g., arrival times of the *S*-wave are assigned *P* velocity, was also examined. In fact, the test has similar results when adding large delays (LPEs) into the arrival times of the *P*-wave, which confirms the superior location stability of the proposed method.

**Figure 3:**
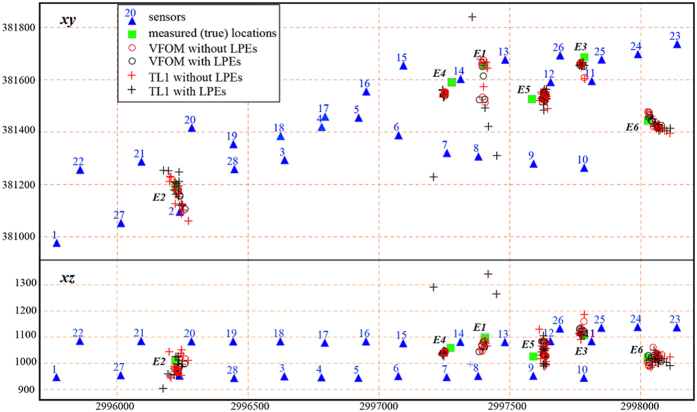
Location uncertainty estimated by the jackknife method. The results from the TL1 method, the best of the four traditional locator methods (see Table 1), are also displayed in the figure for comparison. The black and red circles represent the locations obtained by VFOM using arrival times with and without LPEs, respectively. Similarly, the black and red crosses are the locations obtained by the TL1 locator using arrival times with and without LPEs, respectively. The colour version of this figure is available only in the electronic edition.

We used the jackknife method to estimate the algorithm stability and the location uncertainty of the VFOM. The TL1, the most stable of the traditional methods, was used for comparison (Table 1). We investigated the locations of the two location methods both with and without LPEs, and the results are shown in Fig. 3. The location uncertainty shows that the VFOM can always give stable results with or without LPEs, whereas the TL1 method faces considerable uncertainty when the number of sensors is small and the input data are contaminated by LPEs. For instance, in the VFOM, the uncertainty of E1 changes less than 10 m after the contamination of LPEs, whereas the uncertainty of TL1 increases from 36 m to 218 m. In reality, the in-site microseismic monitoring operation frequently encounters situations in which only a few sensors are triggered, especially for events with very low energy, and these low-energy events often generate signals with a low signal-to-noise ratio, which leads to LPEs. As a consequence, the VFOM is of great practical value for improving the applicability of microseismic monitoring systems.

## Discussion

**Figure 4:**
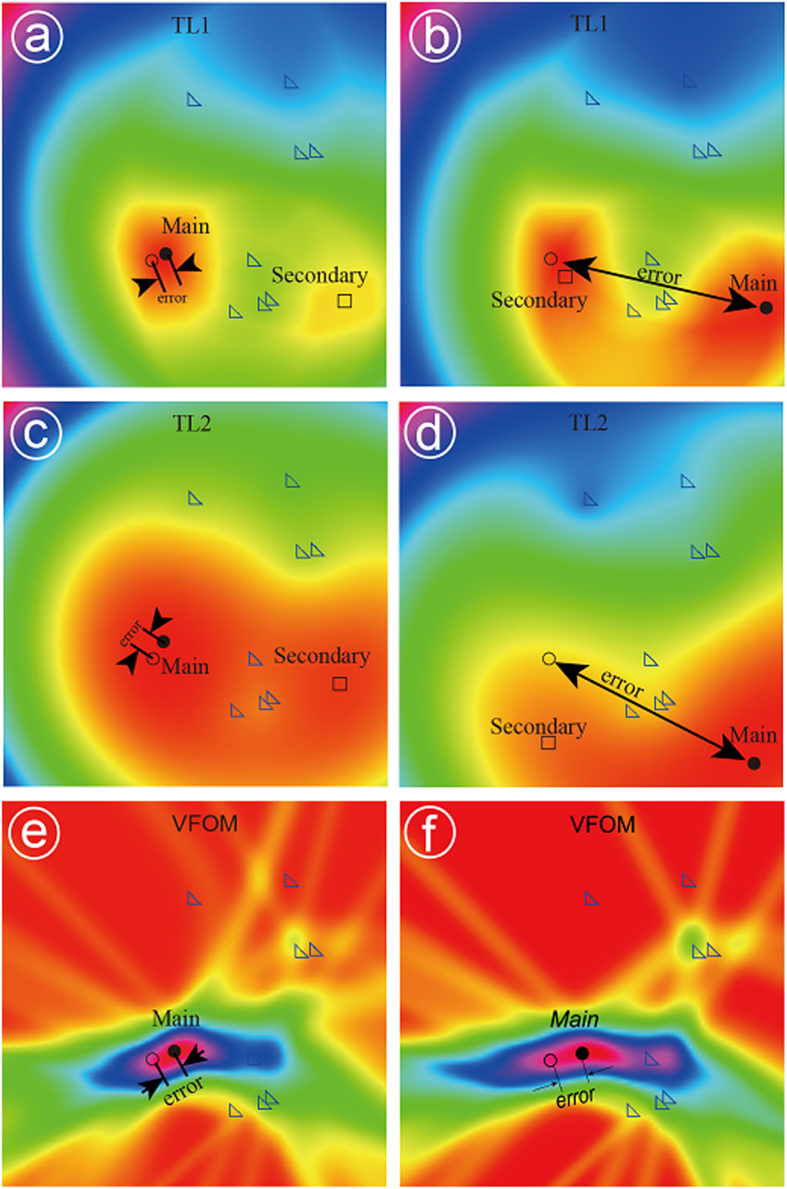
Changes of the objective functions before (a,c,e) and after (b,d,f) LPEs are included. (**a,b**) Are for the TL1 method, (**c,d**) are for the TL2 method, and (**e,f**) are for the VFOM. The circle is the real source, the black dot is the global solution (the main peak), and the square is one of the local solutions (secondary peaks). For traditional locators, the secondary peak may take the place of the main peak after the LPEs are added to the arrival times, whereas this phenomenon rarely occurs with the VFOM.

Many researchers have attempted to improve the solution by introducing a series of searching algorithms with better capacities^30^,^31^. These techniques achieve their purpose by keeping the searching process from converging to local solutions. However, LPEs dramatically change the objective functions of traditional methods. For example, local solutions may turn into the global solution after LPEs are added to the arrival times (Fig. 4a–d). In this case, one would fail to locate the source using any searching algorithm. Figure 4 shows the objective functions of both the VFOM and traditional methods for a 2D source location problem. The objective functions of traditional methods are significantly different before and after the input data are contaminated by LPEs; the global solution shifts from the source to somewhere far away. Moreover, LPE amplitude affects the shift: the larger the LPEs are, the more serious the shift. In contrast, the VFOM has an important advantage for avoiding this impact. As shown in Fig. 4e,f, LPEs remove only the related hyperboloids from the source, and they do not prevent the rest of the hyperboloids from intersecting at the source. This mechanism is why the VFOM can always obtain more stable solutions than traditional methods, even when using input data with LPEs.

A predetermined velocity structure is typically needed for the majority of source location methods using arrival times. It is normal that some error exists between the measured and real velocities in the propagation medium, particularly in media containing fissures, e.g., rock mass. Therefore, we should pay more attention to the velocity sensitivity when a new location method is proposed. Using a series of velocities ranging from 4000 m·s^−1^ to 6000 m·s^−1^, the location errors for a set of synthetic tests were collected. The results showed that the VFOM has a similar velocity sensitivity as traditional methods. It should be noted that all these location methods are less sensitive to velocity errors for the events in the sensor array compared with events out of the array. To obtain a suitable velocity, the production explosion events that are very common in the mining site can be utilized. The positions of these production explosions can be measured as the “real locations” to obtain the best velocity that minimizes the location error. Moreover, the velocity estimated in this way evolves over time in response to the velocity change caused by human activities such as mining. Developing location methods and related work without a pre-determined velocity is another approach to overcoming the issue of velocity errors, which is often treated as an unknown variable^38^,^39^,^40^,^41^,^42^. It will be important to introduce this idea into the VFOM in the future.

Two stopping criteria are available in the VFOM, i.e., *SC-A* and *SC-B*. *SC-A* tends to obtain more stable and accurate results, whereas *SC-B* ensures the success rate of the location program. Synthetic tests show that the errors of the majority of the results obtained by *SC-A* are smaller than 20 m. With regard to *SC-B*, although most of the errors are below 20 m, some locations have errors greater than 50 m. However, *SC-A* can only succeed for 40%–50% of events in the case of 20% LPEs, whereas *SC-B* is able to provide a location for all the events. In summary, both *SC-A* and *SC-B* have advantages and disadvantages, and they can serve as optional choices for practical applications in the presented work.

## Methods

In mathematics, the problem of source location is concerned with solving an over-determined system of equations, i.e., more equations than unknowns^43^,^44^:


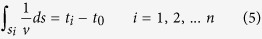


where *s*_*i*_ is the wave propagation path from the source to the *i*^th^ triggered sensor; *v* is the velocity field in space; and *t*_0_ and *t*_*i*_ represent the event’s original time and the arrival time of the wave phase (*P*–wave or *S*-wave) measured by the *i*^th^ sensor, respectively. The system of the classical single-source in homogeneous medium problems is given by


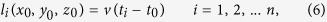


where 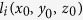 is the distance between the source and the *i*^th^ triggered sensor; 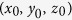
are the coordinates of the source; *t*_0_ and *t*_*i*_ represent the event’s original time and the arrival time of the wave phase (*P*–wave or *S*-wave) measured by the *i*^th^ sensor, respectively; and *v* is the constant propagation velocity (*v*_*p*_ or *v*_*s*_). The most commonly used method to solve the system is to minimize the sum of the square differences between the left side and the right side. Thus, the location problem is transformed into optimizing an objective function. We can obtain the solution by searching the space and time for a point by minimizing the total residual. However, there is a fatal weakness in this method: when there are serious input errors in arrival times (i.e., LPEs, such as mis-picks and outliers), the solution will quite possibly be ruined because all the LPEs contribute to the final location^23^,^24^. This is an important reason why the sources located by many automated microseismic monitoring systems cannot agglomerate into larger clusters in space.

**Figure 5:**
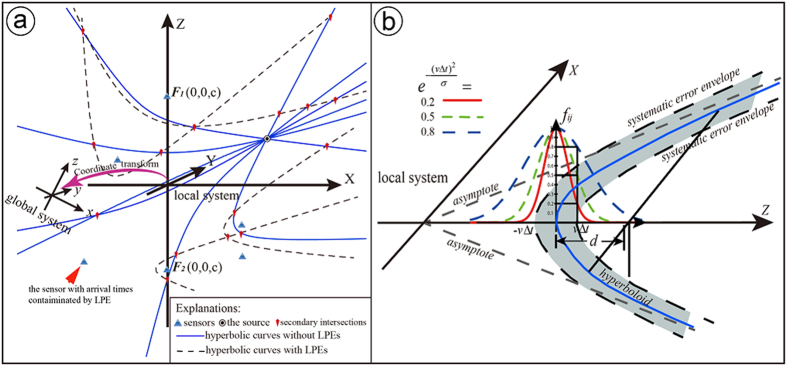
Theoretical explanation of the VFOM. (**a**) The VFOM’s target—the common intersection of the great majority of the hyperboloids (hyperbolic curves in 2D view). The LPE-contaminated arrival time can bias the related hyperboloids but cannot stop the rest of the hyperboloids intersecting at the source. (**b**) Is a virtually established closeness basis for each hyperboloid of (**a**) in the local system. The selection of *σ* determines the shape of *f*_*ij*_. A relatively gentle shape is suggested when lacking confidence in the picking quality, e.g., select a *σ* to let *f*_*ij*_ equal 0.8 at 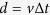 (blue dashed line in Fig. 5b). The stack of the closeness basis generates the largest TCF value at the source.

Instead of minimizing the total residuals between the theoretical and observed arrival times, the VFOM searches the space for the position through which the greatest number of hyperboloids pass (Fig. 5a). The hyperboloid described here is expressed by


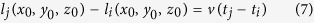


We obtain this expression by subtracting the *i*^th^ equation of Equation. (6) from the *j*^th^ equation. Note that Equation. (7) denotes the geometric definition of a hyperboloid (assuming the right hand is positive) if we treat the source coordinates (i.e., 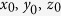) as unknowns. Theoretically, all these hyperboloids in a multi-sensor array should intersect at the source if the arrival times are accurate and the velocity structure is correct. The fact is, unfortunately, that different levels of errors, such as small systematic errors in arrival times, exist even if high-quality picking programs are used. These inevitable errors will cause the hyperboloids to swerve off the source. In consequence, it is difficult to find a point that is on all of the hyperboloids.

To prevent the small errors from misrepresenting the location, we introduce a spatially continuous function called closeness basis (*CB*) to measure the closeness between a point in space and a hyperboloid. Instead of indicating a spatial point on/off a hyperboloid, *CB* describes the closeness between a point and the corresponding hyperboloids by its value at the point. Generally, the source has a relatively high closeness to all the hyperboloids; thus, the sum of *CB*s at the source should reach a higher level than that at other positions. Therefore, by maximizing the sum of *CB*s, we can locate the source. Briefly, we describe the VFOM as follows:

**Step 1:** Establish a *CB* for each sensor pair; its value increases when approaching the corresponding hyperboloid.

**Step 2:** Superimpose all the *CB*s to obtain a spatially continuous function, which is called the total closeness field (*TCF*).

**Step 3:** Search the space for the position by maximizing the *TCF* value with a standard optimization algorithm. 

Thus, we find the position that is close to as many hyperboloids as possible.

### Closeness basis

The *CB* is an overall space function that is able to measure the closeness between a point and its corresponding hyperboloid. If we build a local coordinate system *OXYZ* with *i*^th^ and *j*^th^ sensors symmetrically on the Z-axis, we can rewrite the hyperboloid in a standard form as


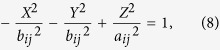


where 
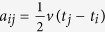
, 
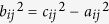
, 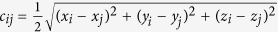. Taking into account small systematic errors (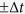), *a*_*ij*_ ranges from 
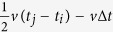
 to 
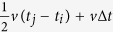
. Correspondingly, the exact position and shape of the hyperboloid change in a certain range (the shadowed area in Fig. 5b). Generally speaking, the closer to Equation. (8) a point is, the greater the likelihood that the point is the source. Here, we establish a virtual field (i.e., *CB*) to quantify the closeness and the possibility. In particular, a feasible *CB* is given by


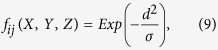


where 
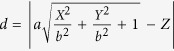
, denoting the Z-direction distance between a point 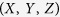 and the hyperboloid of Equation. (8), and *σ* is a constant controlling the shape of the *CB* (the determination of *σ* can be found in Fig. 5b). The value of the *CB* ranges from 0 to 1. With the *CB*’s value increasing, the possibility that the source appears at the position increases.

### *CB*s assemble into the *TCF*

To superimpose the *CB*s in a global system, the relation between local systems and the global system should first be established. In fact, the transformation from the local system *OXYZ* to the global system *oxyz* is the key step in building the *TCF*, and several rotations and transitions can achieve this transformation. For example, an available transformation consisting of two rotations and one translation is given by


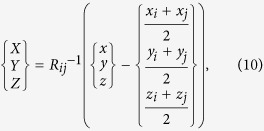


where *R*_*ij*_ denotes a 3 × 3 matrix whose elements are determined by the position of the sensor pair. *R*_*ij*_ combines a y-axis-based rotation and an x-axis-based rotation and is given by


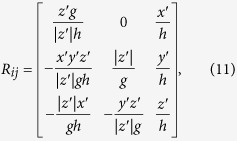


where, 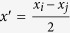

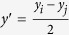

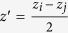

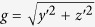
, and 
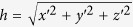
. By using Equation. (10) and Equation. (11),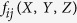 in the local system is transformed to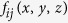 in the global system. Then, we obtain the *TCF* as


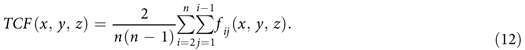


Note that the value of *TCF* also ranges between 0 and 1 because it is the mean of the *CB*s in the global system. If we use both the *P-* and the *S*-wave to locate, *TCF* can be extended as


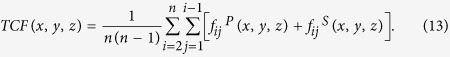


**Figure 6:**
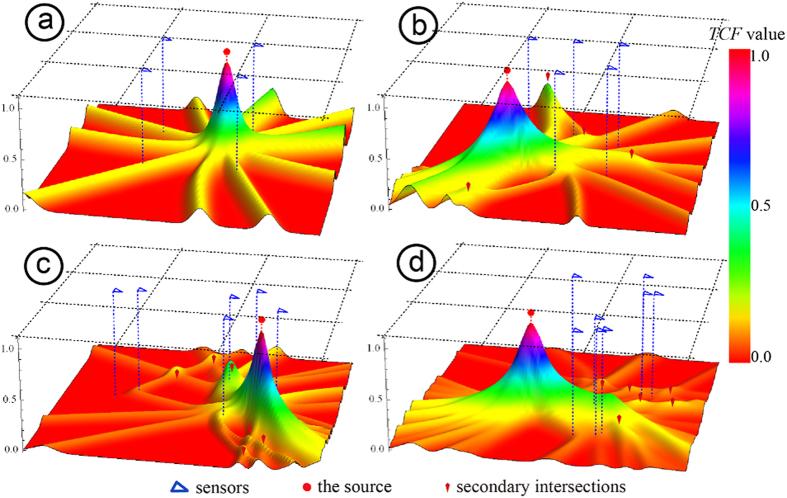
Objective functions (*TCF*) of the VFOM for four 2D location problems. (**a**) Array consisting of 4 sensors, (**b**) array consisting of 5 sensors, (**c**) array consisting of 6 sensors, and (**d**) array consisting of 8 sensors. The array of (**d**) is the same as Fig. 4.

Figure 6 illustrates the *TCF*s of 2D arrays containing 4, 5, 6 and 8 triggered sensors. As shown in Fig. 6, the *TCF* becomes increasingly complex and the number of local optimum solutions (secondary intersections) increases with the rising number of sensors. The rising number of sensors also increases the number of hyperboloids intersecting at the source, which remains the main peak (i.e., the source) standing out against the secondary peaks. This characteristic provides convenience for distinguishing the global solution from local solutions, e.g., setting a threshold as the stopping criterion of the iteration process to estimate whether a solution found is the global solution or not.

### Searching procedure

Many iterative techniques have been used to search for the solution in source location operations, including the grid search technique^19^, the differential evolution algorithm^30^ and the genetic algorithm^31^. The most efficient optimization algorithms are unconstraint local algorithms that utilize the derivative information of the objective function, such as the Quasi-Newton method and nonlinear conjugate gradient methods. Moreover, these algorithms are typically in standard form and easy to obtain. Because of the continuity and differentiability of the *TCF*, we are able to use these standard optimization algorithms as the iterative technique in the VFOM procedure. In general, local optimization algorithms produce solutions that depend on the initial values used. To increase the stability of the solution, we select the best solution after running the iterative process multiple times with different initial values. Thus, stopping criteria are needed to break the iterations. Two stopping criteria are optionally adopted by the VFOM, i.e., stopping criterion *A* (*SC-A*) and stopping criterion *B* (*SC-B*). *SC-A* is a threshold-based criterion that stops the loop with a failure message rather than an unsatisfying solution when no objective value exceeding the threshold is found. A recommended threshold is given by


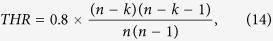


where *k* meets 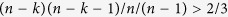 and *n* is the number of triggered sensors. The use of SC-A allows the VFOM to estimate the picking quality. The VFOM may refuse to give the result which is judged to be extremely unreliable when the arrival times contain too many LPEs (e.g., for the recommended threshold, the data with more than 33.3% arrival times containing LPEs will be considered as “unreliable”). Unlike *SC-A*, *SC-B* picks the best solution (with the largest objective value) from the results of multiple trials as its final location. The mechanism of *SC-B* ensures that the VFOM can always obtain the location.

Here we summarize the VFOM location process briefly. The VFOM location process includes three main parts: the initialization, the assembly and the stopping criteria. The assembly process is also divided into two parts: the basis process and the coordinate transform process. *SC-A* and *SC-B* are alternatives for the stopping criteria. By using the searching procedure described above, the VFOM can locate the sources quite efficiently. For example, the whole procedure takes less than 1 second even using a popular personal computer for hundreds of initialize-search processes.

### Uncertainty estimation

The jackknife resampling method is applied to estimate the location uncertainty of the VFOM. The procedure involves repeated relocation, each time subsampling the data by deleting one station at a time. The jackknife method is also employed to estimate the stability of the proposed location method by other researchers^7^,^18^. The standard deviation of Euclidean distances among the locations obtained by the resampled stations of an event is taken as the uncertainties of the event.

## Conclusions

We developed a novel method to locate single-source events from arrival times contaminated by LPEs. This approach, called VFOM, has been verified by both synthetic tests and in-site explosions. The numerical simulations of synthetic tests show that the VFOM is superior to traditional methods for known sources using input data containing different probabilities of LPEs. Furthermore, in the location of explosion events, the VFOM demonstrates its accuracy and stability with both *P* and *P-S* arrivals. Then, we discuss the LPE-tolerant mechanism of the proposed method, which is the resistance to the impact of LPEs on the objective function. The velocity sensitivity analysis shows that the VFOM has a similar sensitivity to velocity errors as traditional methods. In addition, we discuss the properties of the two optional stopping criteria suggested in this paper. The results reveal that the *SC-A* gives more accurate and stable results, whereas the *SC-B* ensures that every event is successfully located. The VFOM is suitable not only for local microseismic location but also for other passive location problems in a homogenous medium such as acoustic source localization.

## Additional Information

**How to cite this article**: Li, X.-B. *et al.* Locating single-point sources from arrival times containing large picking errors (LPEs): the virtual field optimization method (VFOM). *Sci. Rep.* **6**, 19205; doi: 10.1038/srep19205 (2016).

## Figures and Tables

**Table 1:** Location errors of VFOM and traditional methods for the explosion events.

Method	LPEs/Phases used	Absolute location error (m)					
		E1(5)	E2(14)	E3(6)	E4(17)	E5(12)	E6(13)
VFOM	No/P	31.1	49.4	30.8	49.0	48.8	42.8
	Yes/P	44.9	39.1	38.2	47.3	58.5	42.4
	No/PS	37.9	38.3	30.7	46.9	57.4	97.7
	Yes/PS	37.9	38.3	30.7	46.9	57.4	97.7
DL2	No/P	41.3	52.4	39.0	45.5	47.0	96.3
	Yes/P	**>1000**	**222.4**	**>1000**	**192.6**	**307.9**	**379.7**
	No/PS	21.9	48.3	19.3	43.2	51.1	110.5
	Yes/PS	**134.3**	86.0	**>1000**	61.7	**121.6**	**213.2**
DL1	No/P	33.3	40.7	37.2	47.3	46.6	38.5
	Yes/P	**>1000**	34.8	**>1000**	39.0	48.8	**142.6**
	No/PS	15.9	42.8	39.6	45.8	50.1	73.4
	Yes/PS	19.9	39.0	30.3	43.0	51.9	44.0
TL2	No/P	41.3	52.3	39.3	45.6	46.9	95.8
	Yes/P	**>1000**	**222.4**	**>1000**	**192.6**	**307.9**	**379.7**
	No/PS	35.6	94.4	41.8	21.2	83.1	205.8
	Yes/PS	**184.8**	74.8	**>1000**	**84.9**	**184.4**	**263.3**
TL1	No/P	37.7	54.4	33.6	121.2	47.2	22.0
	Yes/P	**306.2**	35.8	**89.4**	93.6	52.1	**81.4**
	No/PS	12.3	91.2	85.1	39.3	68.2	179.6
	Yes/PS	23.0	60.8	95.4	34.2	67.7	214.5
Migration based method	Piking free	131.6	231.4	88.7	257.2	129	215.3
	P arrival time	82.2	45.0	33.5	45.0	47.4	33.5

Numbers in the brackets are the numbers of triggered sensors. Location errors in bold represent those significantly affected by LPEs.
